# Teaching and assessing procedural skills: a qualitative study

**DOI:** 10.1186/1472-6920-13-69

**Published:** 2013-05-14

**Authors:** Claire Touchie, Susan Humphrey-Murto, Lara Varpio

**Affiliations:** 1 The Ottawa Hospital, General Campus, 501 Smyth Road, CPCR 2135 (Box 209), Ottawa, ON K1H 8L6, Canada; 2 Academy for Innovation in Medical Education, University of Ottawa, Faculty of Medicine, Roger Guindon Hall, Room 2034, 451 Smyth Road, Ottawa, ON K1H 8M5, CANADA

**Keywords:** Procedural skills, Objective structured clinical examination, Assessment, Deliberate practice, Simulation

## Abstract

**Background:**

Graduating Internal Medicine residents must possess sufficient skills to perform a variety of medical procedures. Little is known about resident experiences of acquiring procedural skills proficiency, of practicing these techniques, or of being assessed on their proficiency. The purpose of this study was to qualitatively investigate resident 1) experiences of the acquisition of procedural skills and 2) perceptions of procedural skills assessment methods available to them.

**Methods:**

Focus groups were conducted in the weeks following an assessment of procedural skills incorporated into an objective structured clinical examination (OSCE). Using fundamental qualitative description, emergent themes were identified and analyzed.

**Results:**

Residents perceived procedural skills assessment on the OSCE as a useful formative tool for direct observation and immediate feedback. This positive reaction was regularly expressed in conjunction with a frustration with available assessment systems. Participants reported that proficiency was acquired through resident directed learning with no formal mechanism to ensure acquisition or maintenance of skills.

**Conclusions:**

The acquisition and assessment of procedural skills in Internal Medicine programs should move toward a more structured system of teaching, deliberate practice and objective assessment. We propose that directed, self-guided learning might meet these needs.

## Background

Proficiency in procedural skills is an important training objective for Internal Medicine residents according to North American accrediting and certifying bodies [1-3]. In Canada these skills include central venous line and arterial line insertion, endotracheal intubation, thoracentesis, abdominal paracentesis, lumbar puncture and knee joint aspiration [3]. Acquiring these skills requires the development of several different abilities including psychomotor, clinical judgment, communication, decision making, and patient-focused interaction abilities [4]. Direct observation and expert feedback are crucial components to the development of these skills [5], yet appear to be challenging objectives for many programs to meet [6,7].

In modern practice, internists are performing fewer procedures [8] and resident training time must be restricted to respect working hour rules [9]. Given such constraints, clinical exposure alone cannot be solely relied upon to offer procedural skill training that is scaffolded by direct observation and feedback [4].

Various approaches are available to ensure that residents have acquired these skills. For instance, logbooks have a long tradition as tools for assessment of procedural skills. Reports demonstrate that 52.3% of Canadian and 47.8% of American postgraduate programs use case or procedure logbooks as a form of assessment [10,11]. At the time of those studies, logbooks were used at least two times more commonly than simulation. This popularity is likely due, in part, to the ease with which this form of assessment can be implemented. However, logbooks are a means of monitoring the breadth of experiences encountered and of documenting trainee progress. They do not reflect the performance, ensure direct observation or feedback nor ascertain the level of achievement of a trainee [12].

Other innovations aimed at preparing trainees to perform necessary procedural skills are based in the simulation lab. Studies using simulation devices to train physicians in bedside procedures such as central venous line insertion and thoracentesis, crisis resource management, as well as laparascopic and endoscopic skills are well established in the literature [13-20]. Assessment tools to measure proficiency in procedural skills have been created relying on methods such as the McGill Inanimate System for Training and Evaluation of Laparoscopic Skills (MISTELS) and the Imperial College Surgical Assessment Device (ICSAD) [21,22]. The MISTELS is designed to assess technical steps in laparoscopic surgery while the ICSAD has been specifically used for hand motion analysis for performance of technical skills. While useful for highly technical procedural skills, these tools address training and assessment of surgical skills, not of procedures typically done by internists.

Another potential tool for training and assessing residents in procedural skills is the objective structured clinical examination (OSCE). The OSCE has demonstrated validity and reliability in multiple settings [23]. Using multiple stations in an OSCE format was the framework used for the development of the Objective Structured Assessment of Procedural Skills (OSATS) [24,25]. The OSATS is used to assess technical skills through both a procedure-specific checklist and a global rating scale of operative performance. Multiple studies have demonstrated high internal consistency and inter-rater reliability of the OSATS in laboratory multi-station settings and in the operating room [26]. A similar format was also shown to be useful for the assessment of minor surgical skills for clinical clerks and for family medicine residents [27,28]. Structured clinical instructional modules (SCIM) were developed as teaching OSCEs to compensate for difficulty in accessing relevant clinical experience and to provide opportunities to learn about clinical situations that are infrequently encountered by trainees. [29,30]. Additionally, the integrated procedural performance instrument (IPPI) was developed to assess a candidate’s ability to not only demonstrate the technical aspect of a procedural skill, but also the non-technical aspects such as communication, collaboration and professionalism [31]. These stations are logistically more complex and may require more time if included in a more traditional OSCE. Although OSCEs and IPPIs provide opportunities for training and assessment they are expensive and labour intensive.

As this brief review of the literature reveals, research has clearly established that laboratory-based simulation and OSCE frameworks hold great promise as settings for the teaching and assessment of procedural skills for internists. However, while considerable critical attention has focused on evaluating assessment tools, instructional modules, and different teaching and assessment settings, a similar depth of inquiry has yet to delve into the experiences of the trainees who actually face the challenge acquiring procedural skills. Indeed, when it comes to the teaching and assessment of procedural skills, research has carefully examined the delivery side of the question, but has paid less heed to the recipient side. Specifically, little is known about Internal Medicine residents’ individual experiences of 1) learning and acquiring procedural skills proficiency, 2) of practicing these techniques, or 3) of being assessed on their proficiency.

The study described here represents an effort to begin to address this gap. The purpose of this study was twofold. First, it sought to investigate residents’ experiences in the acquisition of procedural skills. Second, it explored resident perceptions of current assessment methods used to evaluate their ability to perform procedural skills. At our school, procedural skills stations were incorporated in a yearly formative Internal Medicine OSCE to evaluate the feasibility and some validity evidence of evaluating the residents using a modified OSATS format [32]. We saw the implementation of this OSCE format to assess procedural skills as an opportunity to ask residents to reflect on learning and assessing procedural skills in general. By using this OSCE as a stimulus for prompting reflection, we hoped to begin to describe some of the basic principles that are common across trainee experiences of acquiring procedural skill proficiency.

## Methods

A fundamental qualitative descriptive approach [33] was the methodological orientation underlying this investigation. Given that the purposes of this study were descriptive in nature, this methodology was employed since it supports inductively identifying and clustering themes from participant statements in order to generate a description of phenomena. In acknowledgement that qualitative research is rarely produced via a single, “pure’ use of a methodology, it should be noted that this study has Grounded Theory overtones [33,34]. This Grounded Theory overtone is realized in the use of constant comparison in this study’s data analysis work. Informed consent was obtained from all the study participants and approval for the study was obtained from The Ottawa Hospital Research Ethics Board.

### Setting

This study was conducted at a Canadian tertiary care teaching hospital. At the time of the study, there was no formal procedural skills curriculum. Internal Medicine residents were expected to acquire procedural skills experience throughout their rotations as opportunities presented themselves. A procedure log was completed by the resident, reviewed by the supervising physician and presented to the program director as a proof of procedure experience. All procedures performed were logged in by the resident regardless of level of supervision. Each procedure type had to be performed at least five times in order to complete the procedural skills requirement. Practice on mannequins for intubation and central line insertion was available once yearly for the residents through a critical care course. The critical care course was mandatory for all the Internal Medicine residents and took place over two days in first year of residency and one day in subsequent years.

All available Internal Medicine residents at this hospital (n=46) participated in an annual formative 10-station OSCE that tested clinical skills. As part of a validation study [32], three procedural skill stations were included as part of the 10-stations: central venous line insertion, endotracheal intubation, and knee joint aspiration. All procedural skills were performed on mannequins (central line mannequin: Simulutions CentraLine Man System; intubation mannequin: Laerdal® Airway Management Trainer; knee aspiration mannequin: Limbs and Things Knee for aspiration) with an expert physician examiner at each station. Stations were designed to test the proficiency of the technical skills. Each procedural skills OSCE station was eight minutes in length followed by two minutes of feedback by the examiner. Additionally, a debriefing session on all OSCE stations was held eight weeks later. The session was held once the station scores were available from the performance examination center for the resident to review. This session included a review of the procedural skills performed on the examination. Data collection for this study occurred immediately following this debriefing session.

### Participants

Participants were recruited on a voluntary basis from the total of 46 residents who had participated in the OSCE. Eighteen residents volunteered to participate in the study. The study participants were representative of the OSCE participants in terms of postgraduate year and overall scores. Participants were assigned to one of the three focus groups in order to purposively distribute participant characteristics which could potentially bias study results [35]. The characteristics that were considered potential biasing factors were: performance ability on the procedural skills stations and post-graduate training year of the participant. The goal of this approach to group composition was to ensure sufficient heterogeneity within the group to stimulate discussion, but also sufficient homogeneity to facilitate comparisons between the focus groups [36].

In the introduction to the focus group, participants were asked to discuss procedurals skills based on their personal experience to date, including but not limited to their recent OSCE experience. Participants were asked to reflect on the entire scope of procedural skills that they were expected to master by the end of training, not just those included in the recent OSCE.

### Data collection

Focus group discussion was selected as the method of data collection since,, as stated by Morgan, “focus groups are useful when it comes to investigating *what* participants think, but they excel at uncovering *why* participants think as they do” [37]. Focus groups allow participants to exchange perceptions of experiences and a wide range of points of views [38]. Given the small participant pool involved in this study, focus group discussions were used in hopes that group dynamics would solicit participant descriptions and reflections that may not have been generated through other data collection methods, such as individual interviews.

Three focus group discussions, each consisting of six participants (thus fitting within the ideal of 5–8 participants) [38], were concurrently held immediately following the OSCE debriefing session. Each focus group was held in a separate meeting room in the hospital. A trained focus group moderator acted as a facilitator of the discussion in each group. The moderators did not attempt to lead or control the group discussion [39]. Instead, each moderator encouraged the participation of all focus group participants, sought to keep any particular participant from dominating the conversation, and endeavored to have conflicting or contrasting opinions heard and discussed by the group. A focus group protocol was used by all moderators (see Focus Group Protocol). The protocol for the focus group was intentionally designed to meet the criteria of effective focus group questions: that is, they were designed to evoke conversation, to be clear, to be short, to be open-ended, and to be one-dimensional [38]. Each focus group was scheduled for an hour, but took an average of 30-minutes likely due to the brevity of the protocol. Moderators took field notes during the focus group discussions to record important themes and discussion trends. At the end of each focus group, the moderator used their field notes to generate a summary of the important themes and discussion trends of that focus group and then reported that summary back to the participants at the end of the focus group session. The moderator asked the group to confirm or to correct the summary. This summary presentation and confirmation process was used as a member checking activity to ensure the confirmability of the study data [40,41].

Each focus group was individually recorded and transcribed. All transcriptions were rendered anonymous during the transcription process. To achieve annonymization, each focus group and resident within the group was assigned a number during the transcription process (e.g.: Resident 3.1 was resident #1 in focus group #3). In this way, no participant identifiers were present in the data set.

### Focus Group Protocol

Three questions were asked of all the focus group participants:

• You recently participated in an OSCE that evaluated your procedural skills. Do you think that this was an acceptable method of testing your skills? Why?

• Can you compare the OSCE testing experience in assessing procedural skills to your current mode of procedural skills assessment? Can you describe the strengths and weaknesses of each?

• Based on your experiences, how do you think procedural skills would best be taught and assessed during your training?

### Data analysis

Data analysis was conducted in several iterative cycles, using a constant comparison technique, focusing on identifying emerging themes in the data and on refining themes and sub-themes into a coding structure [34,42]. In the first analysis cycle, the three focus group transcripts were independently analyzed by three study researchers for emerging trends across all three focus groups. Through iterative cycles of team analysis sessions and individual analysis sessions, the emerging trends were discussed and refined. Common trends from the transcripts were determined through group consensus. This process continued until theme saturation was achieved [34,35]. Confirmability was ensured by maintaining an audit trail of all analytical memos, minutes of the meetings, and revisions to the coding structure. One coder applied the final coding structure to the complete data set, using qualitative data analysis software (Nvivo) to facilitate cross-referencing [43]. Triangulation was built into the study in two ways: 1) using three researchers to ensure investigator triangulation and 2) using three different focus groups and representation of three different resident years to ensure data triangulation [40,44]. While data triangulation via the collection of data by additional methods (e.g. interviews) would have been ideally realized, such inclusion was not logistically feasible.

## Results

Study participants represented all three years of post-graduate Internal Medicine training (8 resident year (R) 1s, 7 R2s, and 3 R3s). Analysis of the focus group data yielded 174 distinct comments from participants, which we grouped into five emergent themes: 1) learning procedural skills; 2) method of assessment; 3) realism of mannequins; 4) direct observation and feedback; and 5) importance of learning procedural skills. Each distinct comment was coded with its surrounding text (2 or 3 sentences) so that the analysis of each participant statement would include consideration of statement contexts. To be designated as a theme, each theme had to include comments from across the three focus groups, and across multiple participants within each focus group. The following reporting of results includes a description of each theme and sample comments from the participants.

### Learning procedural skills

Participants reported that the acquisition of a procedural skill in their day to day activities required both the availability of learning opportunities and the supervision of those opportunities. Participants acknowledged that acquiring procedural skills competency required more practice than they were currently able to obtain stating, for example, “I’m not getting enough experience doing these procedures” (Resident 3.3). The residents recounted that, in their day to day procedural skill learning, they had to actively seek out opportunities to do a procedure, locate someone to supervise them, and felt that they received feedback sporadically. Study participants also acknowledged that they were often supervised not by staff, but by more senior residents: “we learn the procedures from a resident that has been supervised by a senior resident that has been supervised by other senior residents” (Resident 3.5). In addition, the participants who were more senior residents reported that they were expected to fulfill the supervisory role as soon as they were promoted to seniors. One resident commented that “… there needs to be more staff involvement in terms of teaching these procedures when they are available …the minute you are given the senior title and a procedure needs to be done…you are not expecting to just do it but also to teach your juniors” (Resident 1.4). The procedural skills OSCE station was reported as providing an opportunity for learning by many residents. Participants explained that the most important aspect of the OSCE station was the opportunity to learn with experts (staff physicians) who were not only supervising them but also giving them immediate feedback. As one participant explained: “the main benefit of the exam was getting the supervision…as artificial as the feedback was in the exam setting; it is hugely beneficial in terms of what my lack of knowledge is” (Resident 2.1).

Participants also commented on the yearly critical care led course as a means to learn procedures. This offered opportunities to practice central line insertion and intubation in a supervised simulated setting: “…it is run by the ICU staff as well as some Emergency physicians and you kind of go through basic technique on how to intubate, how to do lines, then you run clinical simulations with various case scenarios” (Resident 1.3).

### Method of assessment

At the time of this study, keeping procedure logs was the method used for ensuring competency of procedural skills. Participants commented that this assessment method did not meet their needs. Participants stated that the method of logging procedures did not provide them with insights into whether or not the procedure was properly completed, nor if the procedure was supervised, nor if feedback was given. In their opinion, the logs should not be considered a form of assessment.

The participants found their participation in the procedural skills OSCE particularly rewarding since “[it] is the only evaluation system we have thus far. I mean I would like to see a more complex evaluation system, a little bit more realistic. But for now, I think examining assigned procedural skills [via the OSCE] is a good idea” (Resident 3.3). Even though the participants did not consider the OSCE to be a realistic setting, it did provide some form of assessment. As one participant stated: “it tests whether a person knows sort of the steps and what to do” (Resident 2.3). The participants found the OSCE to be a reasonable and informative system for assessment. There was also strong consensus among participants that they preferred direct observation with immediate feedback. However, the participants could not come to a consensus as to how such observation and feedback could be structured into the program. Finally, the participants also described the OSCE as an acceptable means of ensuring that they possessed adequate technique, but that it could not assess overall performance of the entire skill (i.e. the ability to obtain consent from the patient, the ability to effectively complete the procedure, etc.). The following comment reflects participants’ impression that the OSCE could assess specific aspects of the overall performance of procedural skills: “I thought it was an evaluation of technique and not of ability per se” (Resident 1.4).

### Realism

The residents commented that the simulators did not represent a realistic situation because of some of the physical characteristics of the mannequins. For instance, in discussing the knee aspiration, one participant commented that “all our [simulated] patients are…40 year old males. These mannequins are only young, healthy etc. The knee was the typical knee, you know the 30 year old runner” (Resident 1.5). However, the participants acknowledged that the OSCE was an opportunity to verify their ability to perform the technique. As one participant commented, “…it does check your ability though because …if you cannot get [synovial fluid] on that dummy then you are not going to get any on a real person” (Resident 1.6). Participants reported that this experience was congruent with other stations on an OSCE such as the physical examination station where you may go through the technique of examining without necessarily having positive findings.

### Direct observation and feedback

Participants perceived that when they were observed doing a procedure in a clinical setting, that they were not necessarily getting appropriate feedback. One participant reflected that:

“everyone has sort of different styles of teaching and… of giving feedback… so sometimes you would get excellent feedback and other times, if it is just a resident supervising you, you may not always get as much feedback because they do not want to sort of be confrontational or they want to be encouraging” (Resident 2.3).

The participants reported that more expert staff feedback would be useful to their learning and that they should be able to initiate this when they are on the wards. The participants’ frustration with this lack of expert feedback was evident in their comments:

“I have never had a staff give me feedback at the bedside even if they are watching me.” (Resident 2.1)

“I would say the only time that you are ever really going to get an assessment…if you grab somebody and say: can you watch me do this?” (Resident 1.4)

“Whether it is in the exam setting or whether it is out on the floor… getting staff expert feedback is very beneficial.” (Resident 2.1)

### Importance of learning procedural skills

Participants confirmed that acquiring and being able to complete procedures is a valuable skill. As one resident commented: “as graduates of an Internal Medicine Program, I think it is to be expected that you do graduate having a certain level of proficiency with these skills” (Resident 1.3). The participants stated that it was necessary to have their proficiency in procedural skills evaluated. Finally they suggested that the addition of the procedural skills station in the OSCE would encourage them to be proficient in their procedural skills. They acknowledged that the critical care course served a purpose but that the OSCE offered a further opportunity to practice and demonstrate other procedural skills: “I think it [the OSCE] is beneficial because at least all of us have experienced the course for intubation and central line placement which is helpful because it just kind of reiterates the scenario but personally, I have never aspirated a knee so the fact that I got fluid was kind of funky” (Resident 1.4).

## Discussion

This study provides insights into the perceptions and needs of Internal Medicine residents’ regarding the acquisition and assessment of their procedural skills. Study participants confirmed that it is important to acquire and be able to demonstrate proficiency in procedural skills. They described their present situation predominantly as a resident-directed learning and teaching structure in the clinical setting based on procedure opportunity rather than a structured curriculum. They also expressed a desire for more practice and feedback from direct observation by faculty supervisors. They perceived the OSCE to be an effective formative tool for testing procedural skills that provided a valuable learning opportunity with direct observation and immediate faculty expert feedback on their performance.

While this study provides interesting insights into resident perceptions of the OSCE as a mode of assessment for procedural skills, perhaps the most enlightening comments from participants were their reflections on the acquisition of procedural skills. Participants reported that, at the time of the study, they were responsible for directing the acquisition of and the attainment of proficiency in procedural skills. Participants clearly expressed a need for more directed learning opportunities, feedback, and structured assessments of their procedural skills. Current research confirms that trainees are increasingly participating in such independent learning contexts [45]. Studies are increasingly reporting that self-guided or self-regulated learning can provide effective learning environments for trainees [46-48]. However, as our findings confirm, researchers investigating self-guided or self-regulated learning repeatedly warn that some level of supervision should be maintained [45,49-53], and that complete learner autonomy should not necessarily be the ultimate goal of medical education [54]. Thus, while the resident-directed learning described by our participants may appear to adhere to principles of adult-learning and self-directed learning [55,56], the ad-hoc approach to the acquisition and assessment of procedural skills which is buttressed by limited feedback opportunities is problematic. As our participants report, input from skilled and qualified evaluators (be it in the form of direct observation, feedback or some other form) is an essential component to the success of their self-directed learning processes.

Residents reported that log books were insufficient to ensure proficiency of their procedural skills. The use of log books, however, can keep track of a resident’s procedure skills training, provide an overview of experiences to date, be valuable to monitor progress, and identify deficiencies in training opportunities. In short, log books should not be discounted altogether.

The proliferation of simulation centers in many medical schools provides a potentially safe environment for acquisition and practice of procedural skills. Despite the availability of simulation centers across Canada, a survey of Canadian Internal Medicine residents and program directors conducted in 2008 confirmed that less than 50% of programs were using simulators to teach procedural skills and that 90% of residents were still taught using the traditional “see one, do one, teach one” approach [57]. Two national surveys from the residency accreditation bodies in Canada and the USA, support this notion with reports of simulation use for assessment of less than 30% [10,11].

Our study provides insight into what residents actually do to acquire their procedural skills in the absence of a rigorous faculty-run curriculum. The study also highlights their desire for more direction to frame their self-guided learning. We hypothesize that directed self-guided learning (DSGL) [58] could potentially be used as a means of better supporting Internal Medicine residents’ acquisition of procedural skills. As Brydges recently suggested:

“rather than relying on the technology or the trainee to get it right, a more strategic approach to self-guided learning may be to create conditions so that, even in unsupervised settings, the educator is present through the design and structure of the learning setting. Such a process has been described as directed self-guided learning, and it requires a knowledgeable educator to design practice conditions using validated learning principles” [45].

We hypothesize that DSGL might be able to offer the direction our participants called for, while circumventing the significant challenge of limited supervisor availability. Our qualitative findings help to identify gaps that residents face in acquiring procedural skills and are starting points for addressing these deficiencies.

Based on the principles of transformational change and learning discussed by Kneebone [59] the acquisition of procedural skills should probably be learned in three distinct phases. Firstly, there should be learner-centered practice of the procedural aspect of the skill with a well-defined goal, opportunities for deliberate practice, direct observation and feedback to reach mastery [60]. Although the OSCE may satisfy the need for direct observation and feedback, deliberate and DSGL practice might best be completed in simulation laboratory settings on mannequins. Studies are showing that DSGL provides long-term benefits when compared to instructor-regulator learning in a simulated setting [61]. In addition, simulation-based education (SBE) of procedural skills is valued by residents, demonstrates high clinical competence and retention of skills after mastery training and is translated into better patient outcome [62,63].

The second phase necessary to develop proficiency in procedural skills should again be learner-centered with a patient-focused simulation as described by Kneebone [59]. This setting allows contextual learning of the skill with a simulated patient, providing an opportunity to practice the whole scope of the skill (informed consent, collaboration with other health care professionals, and communication with the patient) in various, potentially challenging situations in a safe environment. This would give the resident a chance to integrate the other skills necessary to properly apply the procedural aspects described above. It would provide a safe environment to allow for potential mistakes which may be beneficial for deeper learning [64]. Assessment of this phase has been demonstrated using the integrated procedural performance instrument [65,66]. The third phase is the integration of the procedure into direct patient care which will require supervision, feedback and assessment of proficiency as well. A tool such as the Direct Observation of Procedural Skills (DOPS) could be used to facilitate this final phase [67].

This study is not without limitations. One limitation is that this study was conducted at a single site and so the perceptions of the residents may be only applicable to one program. Based on the 2010 survey of Canadian Internal Medicine residency programs, our program was not unique since many other programs are not yet taking advantage of simulation and procedural skills curricula [57]. Although this has likely improved since the time of the survey, residents continue to be involved in procedural skills in the clinical setting and may have similar perceptions when faced with the translation of these skills on real patients.

Secondly, the timing of the focus group session eight weeks following the OSCE may have affected the residents’ recall of the use of the OSCE as a teaching and assessment tool. Conducting the sessions immediately after the debriefing session served to remind participants of their experience and provided an opportunity to recall those experiences without the emotional reaction that may have been present immediately after the OSCE itself.

Also, the focus group sample may have been biased by the self-selection of participants; however, more than one third of those who participated in the OSCE agreed to participate in the focus group. Only three senior residents participated in the study which may limit the opinions of this group but this number was proportional to the number of senior residents who participated in the OSCE. In addition, first year residents outperformed the third year residents in some of the procedural skills stations suggesting prior experience with acquiring the procedural skills by the first year residents [32]. Having more senior residents present for the focus groups may have led to further elaboration on the role of senior residents and faculty as supervisors and feedback providers.

Surveys do show that learning procedural skills is essential for the practice of medicine, but that there are few opportunities to practice in the clinical setting and that residents lack confidence in performing these skills [8,20,68]. These findings are confirmed by our residents, and the focus group comments elaborate the reasons and experiences behind these findings. In addition, many themes identified were general and not specific to any particular form of assessment. Thus, we propose that our findings may be transferable to other learning and assessment settings and to other programs.

As procedural skills move beyond the SBE environment and are integrated back into direct patient care, future research documenting the perceptions of residents will be necessary to ensure the transformation change is complete and to assess if similar themes arise in various settings.

## Conclusion

Procedural skills will remain within the scope of practice of internists for the foreseeable future [69]. A serendipitous self-guided approach to acquisition and assessment of procedural skills may not be meeting the needs of postgraduate trainees. Residents participating in this study expressed the importance of direct expert observation with feedback for the assessment of procedural skills. As training programs move towards competency-based training, steps should be taken to provide opportunities for directed self-guided learning for step-wise acquisition of procedural skills including direct observation and feedback. Greater expansion and availability of simulation centers may address some of the gaps identified.

## Abbreviations

OSCE: Objective structured clinical examination; OSATS: Objective structured assessment of procedural skills; MISTELS: McGill inanimate system for training and evaluation of laparoscopic skills; ICSAD: Imperial college surgical assessment device; IPPI: Integrated procedural performance instrument; SCIM: Structured clinical instructional modules; PGY: Post-graduate year; DSGL: Directed self-guided learning; SBE: Simulation-based education; DOPS: Direct observation of procedural skills.

## Competing interests

The authors declare that they have no competing interests.

## Authors’ contribution

All authors contributed to the project conception and design, acquisition of data and data analysis and interpretation. CT drafted the original manuscript. SHM and LV participated in the revisions and final approval of the submitted manuscript.

## Pre-publication history

The pre-publication history for this paper can be accessed here:

http://www.biomedcentral.com/1472-6920/13/69/prepub
